# Supplementary material for: Long-COVID is associated with increased absenteeism from work
Source: PLoS One. 2025 Jun 6;20(6):e0325280. doi: 10.1371/journal.pone.0325280 (PMC12143562; doi:10.1371/journal.pone.0325280)
Supplement: Attachment — Submitted filename: response to reviewers comments.docx [file pone.0325280.s003.docx]

November 30, 2024

Dear Editor and Reviewers,

We appreciate the opportunity to revise our submitted manuscript*, Long-COVID is associated with increased absenteeism from work.* The reviewer's comments were highly insightful and have contributed to strengthening the quality of this paper. Below are the specific reviewer’s comments (in bold) followed by our responses. Newly added parts in the manuscript are highlighted in yellow. Thank you again for taking the time to consider our manuscript.

**Reviewer #1. Overall this is a great paper addressing the outcomes of long covid on missed work days and its financial impact. What I did not see in the article was the consideration of covid vaccine history as vaccination is the primary method to prevent short and long covid symptoms. The study timeline listed of 2021 to 2022 would include the time when vaccines were made available. If MEPS has this data it would be important to include. If not available, I have no further comments. Good job.**

Response: Thank you for the comment. We agree that it is critical to consider whether subjects received the COVID-19 vaccine. Using the following information from the MEPS, we determined the vaccination status of the subjects:

- Ever had a COVID-19 vaccine (Yes/No)
- Ever had a COVID-19 booster shot (Yes/No)

Overall, approximately 83% of the subjects had received the COVID-19 vaccine. Among individuals without long COVID, 83% were vaccinated, compared to 80% of those with long COVID. The difference in vaccination rates between the two groups was not statistically significant (p = 0.124).

Table 1. Percent of subjects with COVID vaccine by long COVID status

|  | Long COVID | |  |  |
| --- | --- | --- | --- | --- |
|  | No | Yes | Total | p-value |
| N | 122,227,065 (92.8%) | 9,458,452 (7.2%) | 131,685,516 (100.0%) |  |
| % of subjects with COVID vaccine | 101,505,619 (83.0%) | 7,530,853 (79.6%) | 109,036,472 (82.8%) | 0.124 |

We accounted for COVID-19 vaccination status in the regression analyses. Tables 2 and 3 below demonstrate the impact of COVID-19 vaccination, as controlled in the regression models.

Table 2. Number of workdays missed by subjects with and without long-COVID

|  | Coefficient | P-value | 95% Confidence Interval | |
| --- | --- | --- | --- | --- |
| Long-COVID | 2.53 | 0.00 | 1.76 | 3.30 |
| COVID vaccine | -0.08 | 0.79 | -0.68 | 0.52 |
| Age category |  |  |  |  |
| 18-30 | Reference |  |  |  |
| 31-40 | -0.16 | 0.71 | -1.00 | 0.68 |
| 41-50 | -0.75 | 0.08 | -1.59 | 0.09 |
| 51-64 | -1.05 | 0.02 | -1.92 | -0.19 |
| ≥65 | 2.49 | 0.18 | -1.18 | 6.16 |
| Female | 1.44 | 0.00 | 1.02 | 1.86 |
| Race/Ethnicity |  |  |  |  |
| Non-Hispanic White | Reference |  |  |  |
| Hispanic | -0.70 | 0.04 | -1.34 | -0.05 |
| Non-Hispanic Black | -0.50 | 0.23 | -1.32 | 0.32 |
| Non-Hispanic Asian | -1.21 | 0.00 | -1.96 | -0.47 |
| Non-Hispanic others | -0.10 | 0.86 | -1.15 | 0.96 |
| Region |  |  |  |  |
| Northeast | Reference |  |  |  |
| Midwest | -0.11 | 0.76 | -0.82 | 0.60 |
| South | 0.17 | 0.64 | -0.56 | 0.91 |
| West | 0.81 | 0.03 | 0.08 | 1.53 |
| Type of health insurance |  |  |  |  |
| Private insurance | Reference |  |  |  |
| Medicaid | 0.74 | 0.13 | -0.21 | 1.68 |
| Medicare | -2.90 | 0.00 | -4.10 | -1.70 |
| No insurance | -0.85 | 0.06 | -1.75 | 0.04 |
| Marital status |  |  |  |  |
| Married | Reference |  |  |  |
| Widowed/divorced/separated | 0.59 | 0.09 | -0.08 | 1.26 |
| Never married | -0.26 | 0.37 | -0.83 | 0.31 |
| High school graduate | -0.65 | 0.13 | -1.49 | 0.19 |
| Hypertension | 0.47 | 0.14 | -0.15 | 1.08 |
| Diabetes | 0.49 | 0.29 | -0.42 | 1.40 |
| Asthma | 1.51 | 0.00 | 0.73 | 2.30 |
| High cholesterol | 0.39 | 0.14 | -0.12 | 0.89 |
| Cancer | 0.89 | 0.08 | -0.11 | 1.88 |
| Arthritis | 2.11 | 0.00 | 1.40 | 2.82 |
| Physical limitation | 2.51 | 0.00 | 1.46 | 3.56 |
| Cognitive limitation | 2.54 | 0.00 | 0.94 | 4.13 |
| Current smoker | 0.66 | 0.12 | -0.16 | 1.48 |

The COVID-19 vaccine variable is not statistically significant in the regression analysis (coefficient = -0.08, p = 0.79). Controlling for the COVID-19 vaccine variable does not affect the coefficients of the other control variables in the regression. Similarly, the coefficient of the long COVID variable remains consistent between regressions with and without the COVID-19 vaccine variable. Specifically, in the regression without the vaccine variable, the coefficient for long COVID is 2.54 (p < 0.01), while in the regression with the vaccine variable, it is 2.53 (p < 0.01).

Table 3 below presents the results when accounting for COVID-19 infection status categories (no infection, history of COVID-19 infection, and long COVID). In this analysis, the COVID-19 vaccine variable remains statistically insignificant (coefficient = -0.10, p = 0.74). Adding the vaccine variable does not change the coefficients of the COVID-19 infection status variables. For instance, without the vaccine variable, the coefficient for history of COVID-19 infection is 1.90 (p < 0.01), and for long COVID, it is 4.05 (p < 0.01). When the vaccine variable is included, the coefficients are nearly identical: 1.89 (p < 0.01) for history of COVID-19 infection and 4.02 (p < 0.01) for long COVID.

Table 3. Impact of COVID-19 on missed workdays: Comparison between no infection, history of COVID-19 infection, and long-COVID

|  | Coefficient | P-value | 95% Confidence Interval | |
| --- | --- | --- | --- | --- |
| COVID-19 infection status |  |  |  |  |
| No infection | Reference |  |  |  |
| History of COVID-19 infection | 1.89 | 0.00 | 1.39 | 2.39 |
| Long-COVID | 4.02 | 0.00 | 2.85 | 5.18 |
| COVID vaccine | -0.10 | 0.74 | -0.70 | 0.50 |
| Age category |  |  |  |  |
| 18-30 | Reference |  |  |  |
| 31-40 | -0.17 | 0.69 | -1.01 | 0.67 |
| 41-50 | -0.71 | 0.09 | -1.53 | 0.11 |
| 51-64 | -0.88 | 0.05 | -1.74 | -0.02 |
| ≥65 | 2.41 | 0.18 | -1.13 | 5.96 |
| Female | 1.40 | 0.00 | 0.98 | 1.81 |
| Race/Ethnicity |  |  |  |  |
| Non-Hispanic White | Reference |  |  |  |
| Hispanic | -0.66 | 0.05 | -1.32 | -0.01 |
| Non-Hispanic Black | -0.25 | 0.57 | -1.12 | 0.62 |
| Non-Hispanic Asian | -1.09 | 0.01 | -1.85 | -0.33 |
| Non-Hispanic others | -0.04 | 0.93 | -1.07 | 0.99 |
| Region |  |  |  |  |
| Northeast | Reference |  |  |  |
| Midwest | -0.09 | 0.80 | -0.79 | 0.61 |
| South | 0.29 | 0.45 | -0.46 | 1.04 |
| West | 0.82 | 0.02 | 0.11 | 1.53 |
| Type of health insurance |  |  |  |  |
| Private insurance | Reference |  |  |  |
| Medicaid | 0.83 | 0.08 | -0.10 | 1.76 |
| Medicare | -2.63 | 0.00 | -3.88 | -1.38 |
| No insurance | -0.59 | 0.20 | -1.50 | 0.32 |
| Marital status |  |  |  |  |
| Married | Reference |  |  |  |
| Widowed/divorced/separated | 0.65 | 0.06 | -0.02 | 1.32 |
| Never married | -0.17 | 0.57 | -0.73 | 0.40 |
| High school graduate | -0.75 | 0.09 | -1.62 | 0.12 |
| Hypertension | 0.49 | 0.12 | -0.13 | 1.12 |
| Diabetes | 0.47 | 0.31 | -0.44 | 1.38 |
| Asthma | 1.44 | 0.00 | 0.67 | 2.21 |
| High cholesterol | 0.36 | 0.17 | -0.15 | 0.87 |
| Cancer | 0.83 | 0.10 | -0.16 | 1.82 |
| Arthritis | 2.03 | 0.00 | 1.31 | 2.75 |
| Physical limitation | 2.50 | 0.00 | 1.43 | 3.58 |
| Cognitive limitation | 2.54 | 0.00 | 0.92 | 4.15 |
| Current smoker | 0.83 | 0.06 | -0.03 | 1.69 |

Additionally, we included interaction terms between long COVID status (no COVID infection, moderate COVID infection, and severe COVID infection) and COVID-19 vaccine status. However, the interaction variables were all statistically insignificant, and the main results for long COVID status remained unchanged.

Since the inclusion of COVID-19 vaccine status did not alter the results, we propose to retain the original reported results in the manuscript. However, we have added the following information to the Results section:

"Another sensitivity analysis included COVID-19 vaccine status (i.e., received a COVID-19 vaccine or booster shot—Yes/No) in the regressions, as vaccination may reduce the risk of short- and long-COVID symptoms. Among the subjects, 83% without long COVID had received the COVID-19 vaccine, compared to 80% of those with long COVID. The difference in vaccination rates between the two groups was not statistically significant (p = 0.124). Furthermore, the COVID-19 vaccine status variable in the regression analysis was not statistically significant and did not affect the main findings.”

**Editor Comments:**

**A very well written manuscript exploring the economic impact of long Covid due to loss of productivity, using the population data available in MEPS, a representative sample of the U.S. population. In agreement with the reviewer's observation, an individual with subject matter expertise, it would be interesting to factor in the impact of the Covid vaccine on long Covid since the dataset used was from 2021-2022 at a time when the vaccines were freely available. If MEPS captured this data and you still have access to the dataset and are willing to factor in the use of COVID vaccines in your analysis, that would further enrich the results and make them more meaningful. In the absence of the datasets or your lack of time to take on the additional work involved, a statement explaining the absence of any reference to the vaccines in the manuscript would be needed.**

Response: Please refer to the responses above. We hope that we have adequately addressed your comments.

**Journal Requirements:**

**When submitting your revision, we need you to address these additional requirements.**

**1.Please ensure that your manuscript meets PLOS ONE's style requirements, including those for file naming. The PLOS ONE style templates can be found at**

[**https://journals.plos.org/plosone/s/file?id=wjVg/PLOSOne_formatting_sample_main_body.pdf**](https://journals.plos.org/plosone/s/file?id=wjVg/PLOSOne_formatting_sample_main_body.pdf) **and**

[**https://journals.plos.org/plosone/s/file?id=ba62/PLOSOne_formatting_sample_title_authors_affiliations.pdf**](https://track.editorialmanager.com/CL0/https:%2F%2Fjournals.plos.org%2Fplosone%2Fs%2Ffile%3Fid=ba62%2FPLOSOne_formatting_sample_title_authors_affiliations.pdf/1/010f01934c5975de-2efddbc9-8af7-4640-98cf-ccd5e8327003-000000/GXbhuT4P9UnrwnhORh9ICdHZKAfUvvmV8D0HqyCj7vM=186)

Response: We double-checked the journal’s style requirements and confirmed that the manuscript aligns with them.

**2. Your ethics statement should only appear in the Methods section of your manuscript. If your ethics statement is written in any section besides the Methods, please move it to the Methods section and delete it from any other section. Please ensure that your ethics statement is included in your manuscript, as the ethics statement entered into the online submission form will not be published alongside your manuscript.**

Response: We removed the following ethics statement from the end of the manuscript:

**Ethics statement**: Since the MEPS data used in this study are publicly available, Internal Review Board (IRB) review was not required.

**3. We note you have included a table to which you do not refer in the text of your manuscript. Please ensure that you refer to Table 1,2 and 3 in your text; if accepted, production will need this reference to link the reader to the Table.**

Response: We added Table 1 on page 7, Table 2 on page 9, and Table 3 on page 10 to the text.

**4. Please review your reference list to ensure that it is complete and correct. If you have cited papers that have been retracted, please include the rationale for doing so in the manuscript text, or remove these references and replace them with relevant current references. Any changes to the reference list should be mentioned in the rebuttal letter that accompanies your revised manuscript. If you need to cite a retracted article, indicate the article’s retracted status in the References list and also include a citation and full reference for the retraction notice.**

Response: We reviewed the references, and all are accurate and complete.

Thank you once again for taking the time to review the manuscript!
