# Supplementary material for: Long-COVID is associated with increased absenteeism from work
Source: PLoS One. 2025 Jun 6;20(6):e0325280. doi: 10.1371/journal.pone.0325280 (PMC12143562; doi:10.1371/journal.pone.0325280)
Supplement: Attachment — Submitted filename: PONE-D-24-37720_review.docx [file pone.0325280.s002.docx]

**Long-COVID is associated with increased absenteeism from work**

**Review**

This paper aims to study the association between long-COVID and workplace absenteeism in workplace using a representative sample of the U.S. population.

What I would like to point is the lack of definition of the covariate “cognitive impairment”, since psichiatry and neurological disorders can present symptoms like COVID-19 long syndrome. (https://www.nature.com/articles/s41591-024-03309-8)

Other consideration is the severity of Covid desease considered in the groups analysed, since it can present different spectrum of clinical recover / post-Covid syntoms.

Finally, i would like to know if the Authors considerate the mediation effect of cognitive impairment in the outcome analysed, since is known that symptons of cognitive impairment due to Covid-19 infection could per se be associated with absenteeism in workplace.
